# The spread of the cult of Asclepius in the context of the Roman army benefited from the presence of physicians: A spatial proximity analysis

**DOI:** 10.1371/journal.pone.0256356

**Published:** 2021-08-20

**Authors:** Tomáš Glomb

**Affiliations:** Department of Archaeology, History, Cultural Studies and Religion, University of Bergen, Bergen, Norway; Universidad Rey Juan Carlos, SPAIN

## Abstract

The article applies a GIS based approach to the study of the spread of the cult of Asclepius, the Greco-Roman healing god, during the Roman period. It explores the role of soldiers and physicians in the spatial dissemination of the cult along the transportation network of Roman roads in the border provinces of Britannia, Germania Superior and Inferior, Raetia, Noricum, Pannonia Superior and Inferior, Moesia Superior and Inferior, and Dacia. These provinces were selected as a suitable area for quantitative GIS exploration because they were all on the outer border of the Roman Empire, had a significant military presence, and there is a representative amount of inscriptions attested that can be used as proxies for the spatial occurrence of the three measured variables: the cult of Asclepius, Roman soldiers, and Roman physicians. After establishing by means of spatial proximity analysis that the cult of Asclepius occurred frequently in the context of the Roman army, the article proposes and quantitatively evaluates a more specific hypothesis; i.e., that the spatial occurrences of Roman physicians in inscriptions are a relevant predictor for the spatial occurrences of the worship of Asclepius in the environment of the Roman army because of the shared focus between physicians and the cult of Asclepius—health and medicine. The highly significant results of the statistical analysis reveal a positive trend in the spatial relationships between Roman physicians and the worship of Asclepius in the context of the Roman army in the majority of provinces of interest, thus supporting the proposed hypothesis. The results presented in the article demonstrate the potential of the GIS approach in testing assumptions produced by traditional scholarship and in nuancing our understanding of a specific process of cultural spread.

## Introduction

Asclepius was one of the most popular healing deities of the Greco-Roman world. Originally from Greece, the cult of this mythical son of Apollo was officially invited to Rome in 293 BCE and became firmly rooted in Roman society as a patron deity of health [1–5]. The uniqueness of Asclepius’ cult, when compared to other Graeco-Roman cults, lies in 1) the promise of a close relationship between the god and his supplicant, because the supplicant expected to meet the god in their dreams during the temple rituals of incubation; 2) the patients’ deep expectation of being healed; and 3) a non-exclusive membership for people across socio-economic groups [1, 3, 6]. A potential fourth point argued here is that the cult of Asclepius could have had a special appeal among Roman soldiers, as they were often in the need of medical care due to combat wounds or inhospitable environments at the border provinces of the Empire.

In the first centuries CE, the “romanized” version of the cult of Asclepius, represented in material culture mainly by altars and temples bearing Latin inscriptions, spread as far as North Africa to the south, Hadrian’s wall to the north, Spain to the west, and the shores of the Black Sea to the east [5]. Although recent works on the cult of Asclepius during the time of the Roman Empire exist (see e.g. [1, 2]), these approach the spread of the cult by traditional historiographical methods and base generalizing conclusions on the interpretation of selected pieces of archaeological or literary evidence. While this has clear merits in situating the cult in its societal context, the potential for disentangling the more complex interactions involved in the dissemination of religious cults is limited. This article introduces a formal GIS approach as a supplementary tool for shedding more light on the mechanisms and factors involved in this process of cultural dissemination.

In this study, the method of spatial proximity analysis was applied to quantitatively test the established hypothesis that soldiers were one of the major factors in the dissemination of ancient cults [7–11]. This hypothesis has recently been examined in connection to the cult of Asclepius in the Roman period by Ghislaine van der Ploeg. She found the notion plausible, and pointed to evidence of an explicit interest in the cult by Roman soldiers, such as inscriptions dedicated to Asclepius and an inscription from Novae (Moesia, modern Bulgaria) dedicated to Asclepius for the good health of an entire legion [2]. Based on this evidence, there is little doubt that the cult of Asclepius was popular among soldiers. Such case studies, however, have little potential to reveal further potential patterns in the relationship between soldiers and the cult of Asclepius.

**Fig 1:**
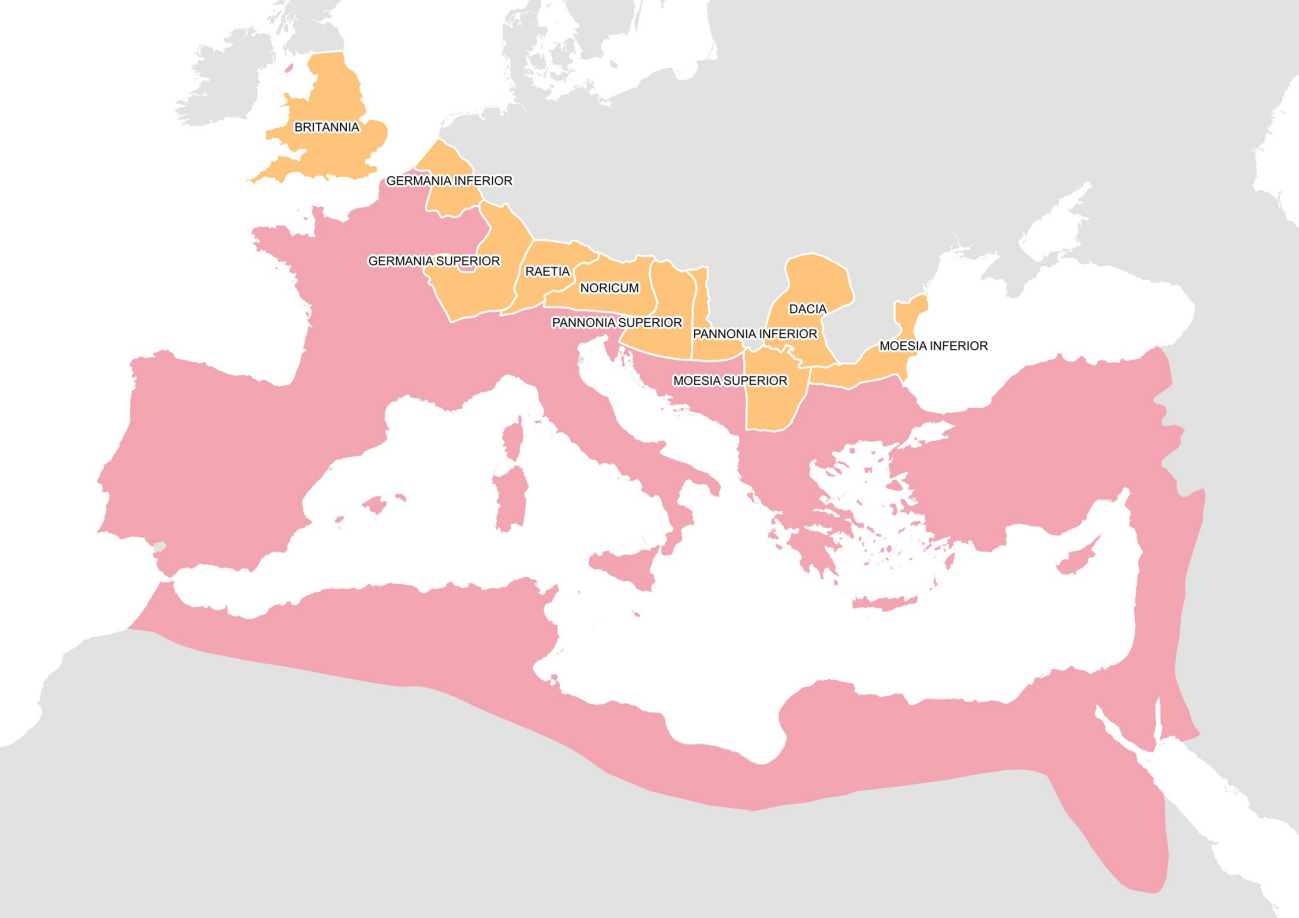
The area of interest. The provinces of interest are in orange, the Roman Empire in red. Sources: Natural Earth [31]; Ancient World Mapping Center (AWMC) [32]—Reprinted from AWMC database of shapefiles [32] under a CC BY license, with permission from AWMC, original copyright 2012.

Recently, the combination of established methods and formal approaches (e.g., network analysis, GIS, agent-based modeling, quantitative textual analysis) has demonstrated significant potential for the historiography of the ancient Mediterranean (and beyond) [8, 10–21]. The present article follows this academic trend and reveals, by means of a GIS proximity analysis based on the platform of Roman transportation road network, specific spatial patterns in the worship of Asclepius in the military environment in the provinces along a portion of the border of the Roman Empire leading from Britannia to Moesia Inferior (i.e., Britannia, Germania Superior and Inferior, Raetia, Noricum, Pannonia Superior and Inferior, Dacia, and Moesia Superior and Inferior, Fig 1). These provinces were selected as the area of interest because 1) they share a specific outer border of the Roman Empire, 2) they had a significant military presence in the first three centuries CE [22–28], and 3) they offer representative data for spatially approximating the worship of the cult of Asclepius and the dissemination of military presence [29]. The selected provinces thus constitute a relevant and sufficiently coherent area for exploring both regional and extra-regional patterns. The period of interest is that of the Roman Empire, i.e., a time of intensive efforts for Roman expansion. However, the amount of material evidence for the worship of Asclepius and the presence of soldiers in the selected provinces reaches its peak in the 2nd and 3rd centuries CE and, therefore, the research is mainly representative of this timeframe [2, 29, 30].

Finally, this article reveals a statistically significant spatial pattern in these provinces between the worship of Asclepius in the military environment and the occurrence of physicians in inscriptions, thus inviting further focus on this layer of the social milieu of the Roman army. To further support the validity of the results of this research, the present article compares the patterns in the spatial dissemination of the cult of Asclepius with other cults such as the cult of Apollo, Minerva, and Jupiter.

## Methods

### Study design–an overview

The spatial relationships between the Roman army, Roman physicians, and the cult of Asclepius within the area of interest were explored by testing two hypotheses: 1) Roman soldiers played an important role in the spread of the cult of Asclepius, and 2) presence of Roman physicians was beneficial for the spread of the cult of Asclepius. The hypotheses were tested in this order since testing of the second is dependent on the validity of the first. No permits were required for the described study, which complied with all relevant regulations.

The role of Roman soldiers in this cultural transmission was analyzed by measuring and evaluating spatial proximity between the locations of Roman military presence and places of worship of Asclepius as approximated by literature and inscriptions in the area of interest. The rationale behind the analysis is that if the cult and military proxies frequently co-occurred spatially, this indicates that the soldiers played a positive role. The data for evaluating spatial proximity were collected in a GIS environment as geographical distances of the shortest paths between the proxies for military presence and the worship of Asclepius on the Roman transportation road network. For contextualization and comparison purposes, proxies for the spatial worship of Apollo, Minerva, and Jupiter were incorporated in the analysis (rationale for the selection discussed in the section Variables). The distances were measured in two ways: 1) from places of military presence to the nearest cult sites, and 2) from cult sites to their nearest place of military presence. This way it was possible to explore the overall spatial overlap between military presence and worship of individual deities, and the extent to which the cults appeared in military contexts. To further explore the intensity of military worship of the selected cults, a methodologically identical spatial proximity analysis was conducted for Roman settlements in the area of interest representing an alternative and competing set of locations for measuring distances to the nearest cults and vice versa. The distances were evaluated by descriptive and exploratory statistics (i.e., Mann-Whitney U test, Kruskal-Wallis H test) to help determine the differences between the cults and whether the cults were significantly more attached to military contexts than to Roman settlements.

The second hypothesis exploring the impact of Roman physicians on the spatial dissemination of the cult of Asclepius in the military environment was tested by measuring and evaluating the shortest geographical distances on the transportation network from each unique military position to its nearest location of 1) an inscription attesting presence of a Roman physician, and 2) an inscription attesting the worship of Asclepius. The measurements were repeated in this fashion for the spatial proxies for the worship of Apollo, Jupiter, and Minerva. The distances from military positions to proxies for Roman physicians and the cults were evaluated by Spearman’s rank correlation coefficient to determine whether there is a significant correlation between these spatial relationships which would suggest a positive role of the presence of Roman physicians on the spread of a particular cult in the military environment.

Although the Roman road network platform is essential for the analytical part of this study, the overall approach is conceptualized as a spatial proximity analysis rather than network analysis. The presented study does focus on relational phenomena structured by a network, however, the relationships here are analyzed in terms of geographical proximity on the network and not by the methods of network science such as centrality measures that identify important nodes in a network graph. Despite this differentiation presented here for the purpose of methodological clarity, the two approaches are mutually compatible and often combined in historiographical or archaeological studies (for discussion see e.g., [33–39], for studies using network approaches in geographical or transportation context see e.g., [11, 14, 15, 21, 40–42]).

The selection and operationalization of the transportation network, variables, as well as specific methodological steps are discussed in more detail in following sections.

### Network

The people living in the provinces of interest and Roman soldiers stationed there were the potential carriers of the cultic practices. The livelihood and movement of these people were significantly restrained by the physical environment, and they were not able to travel freely across the provinces as if they were a homogenous terrain. Instead, the mobility and social connections in the area of interest depended on a transportation network consisting of Roman roads. Moreover, Roman soldiers were a highly mobile part of society, often sent to distant lands from more central territories of the empire [23, 24, 30]. Therefore, to explore the spatial relationships between the places of worship and the presence of the Roman army it is appropriate to conduct the analyses using the platform of this transportation network.

The Stanford geospatial network model of the Roman world (ORBIS [43]) provides a quality transportation network platform for researchers focusing on mobility in the ancient Mediterranean. It allows researchers to calculate the fastest, shortest, or cheapest connections between selected cities based on the distance, mode of transport used, and seasonality. The problems with ORBIS with respect to the research presented in this article are scale and resolution. Although ORBIS is based on a thorough application of ancient and modern sources, it is primarily constructed to map long-distance connections and the model does not provide adequate density of Roman roads at the level of individual provinces.

**Fig 2:**
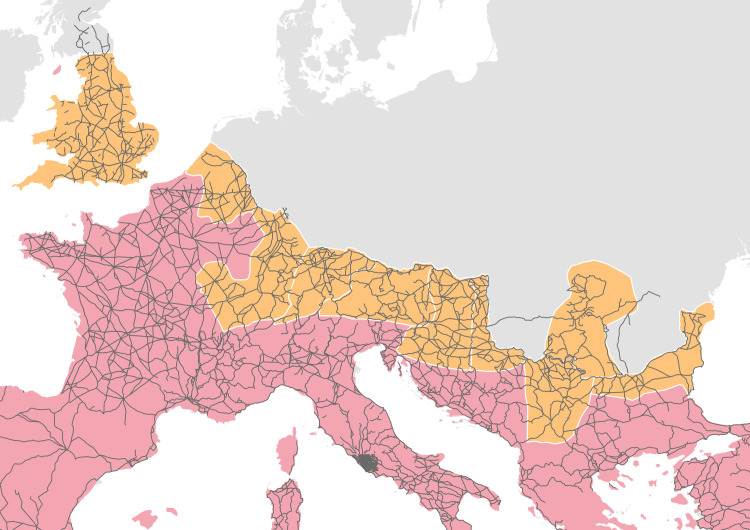
Transportation network of Roman roads. Sources: Natural Earth [31]; Ancient World Mapping Center (AWMC) [32]—Reprinted from AWMC database of shapefiles [32] under a CC BY license, with permission from AWMC, original copyright 2012.

Therefore, the dense network of attested Roman roads digitalized and geocoded by the Ancient World Mapping Centre (AWMC [44]) based on the *Barrington Atlas of the Greek and Roman World* [45] was used as a more accurate and realistic transportation network in the area of interest (Fig 2). The trajectories and intersections of these roads were then validated in GIS software by reconnecting loose ends due to minor errors in the coordinates of the original dataset. This network of Roman roads was then used in the subsequent analyses as a platform for defining relationships between the variables based on their mutual distances within this network.

### Variables

The examined variable is the spatial pattern of the worship of Asclepius and its relationship to the Roman army. The practice of naming the god in inscriptions from the relevant period and region is conceptualized as a suitable proxy for the spatial dissemination of this cult. A significant portion of the evidence for the worship of deities in Roman times comes from inscriptions, as they document a range of levels of interaction, from dedicating a temple, altar or statue to fulfilment of vows, over a period of hundreds of years [46]. The selected provinces had a high military presence and the habit of making inscriptions (primarily in Latin) is richly attested in the Roman military environment, even in areas without any previous comparable tradition, which is particularly the case for the provinces in question [23, 30, 47, 48]. The Roman inscriptions from the area of study are attested in representative numbers and collected in the Epigraphic Database Heidelberg (EDH [29]). Attributes of the EDH inscriptions include a unique ID, name and geographical coordinates of location, provinces of origin, text of inscription, social information (e.g., whether military personnel are mentioned, or a person from the senatorial order, etc.), date ranges (as a *terminus post* and *ante quem*), type of inscription (e.g., votive inscription, epitaph, etc.), and corpus references. These attributes allow for the quantification of the relevant variables. To approximate this level of interaction with the cult of Asclepius, the inscriptions were collected based on the following rules: the Roman Empire was selected as the historical period (i.e., date range 27 BCE—476 CE); the geographic coordinates or the findspot are attested; the text of the inscription includes the name of the god Asclepius in different noun-cases and alternative forms (e.g., Aesculapius, Esculapius and their noun-cases) and excluding personal names (such as Asclepiades or Asclepiodotus, or sometimes even Asclepius). The amount (N) of this variable in the provinces of interest is 124 [49].

The military presence was approximated in two ways. First, the long-term (i.e., active for more than a century) bases of Roman legions were geocoded based on the literature, as a proxy for an intensive and long-term presence of Roman soldiers at a given location (N = 19) [49–53]. Second, to have a more dense and granular proxy for a military presence in the area of interest, EDH inscriptions from the Roman Empire period with coordinates and with the “military personnel” attribute were collected. Additionally, as the focus of this article is on a specific practice of making inscriptions to deities, only the inscriptions of military personnel that were classified as “votive inscriptions” were included in the analysis (N = 1937) [49]. These represent a suitable proxy for geographical dissemination of soldiers involved in this practice.

As Asclepius was the god of medicine, and there are altars dedicated to Asclepius attested in the *valetudinarium* (i.e., Roman military hospital) in Novae (see e.g., EDH no. HD043500, HD043503, HD043504), an additional variable was selected to examine a potential and more specific relationship between the worship of Asclepius and the military social environment: in this case, Roman physicians. Particularly, the research explores how the practice of naming Asclepius in inscriptions relates to the presence and activities of physicians attested in inscriptions. The quantitative exploration of this factor has the potential to bring more nuance and a new perspective to our knowledge of the spreading dynamics of the cult of Asclepius. To capture this variable, inscriptions from the period and area of interest containing mentions of physicians were selected as the most suitable proxy and stored as a dataset (N = 44) [49]. Physicians appearing in the EDH inscriptions from the area of interest belonged almost exclusively to the social environment of the Roman army, as is attested by their spatial and temporal overlap and frequent attributes co-occurring next to the word *medicus* (physician) and its varying noun-cases in the inscriptions, such as *ordinarius* and *miles* (army physicians), *legionis* (physicians of the legion), or *cohortis* (physicians of the cohort). These attributes reflect the reality that military physicians were part of the specialized and organized medical care in the Roman army [1, 54–56]. Such organization of medical services in the military was necessary due to the expansion of the Empire after Augustus. In the context of the Empire’s expansionist policy, it was no longer possible for civilian physicians in cities to provide medical treatment for soldiers stationed in remote camps and garrisons [1, 57]. The archaeological evidence for *valetudinaria* on the borders of the Roman Empire is another indicator of the division between civilian and military medical treatment in remote locations [56, 58]. In order to at least partially estimate the possible social or demographic proportionality of Roman physicians to Roman soldiers in the area of interest, the number of epitaphs naming the occupation of *medicus* was compared to the number of epitaphs attributed to military personnel based on EDH. In 8 out of 10 provinces, the epitaphs of Roman physicians compare to approximately 0.5–1.5% of the number of epitaphs connected to military personnel. The exception is Raetia with 3,5% and Noricum with no epitaphs for Roman physicians. The literature gives an estimate of 1 physician per 500 troops and ca 500–800 physicians for the Roman army in the 2nd century CE [2, 59].

**Fig 3:**
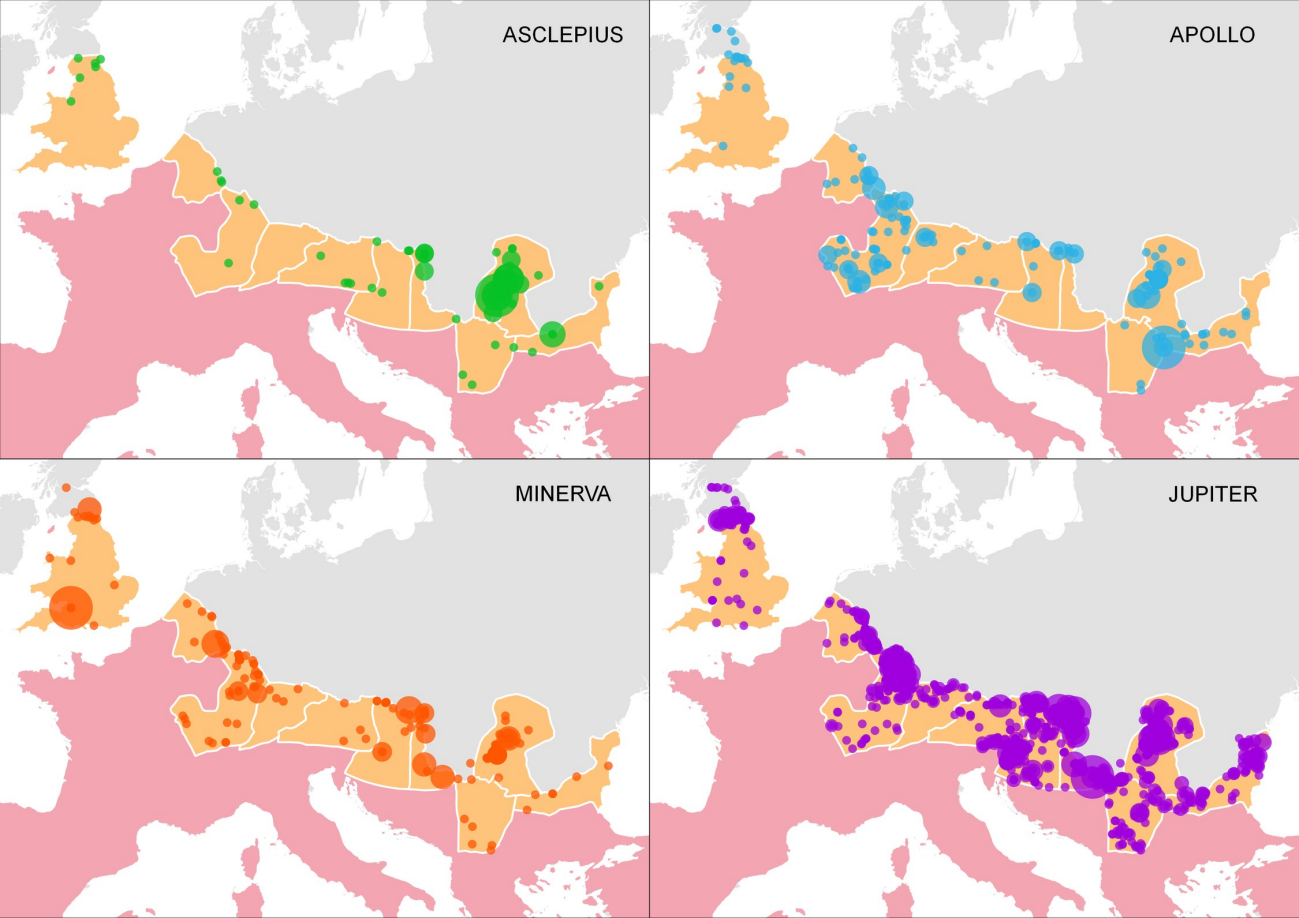
Inscriptions with Asclepius, Apollo, Minerva, and Jupiter from the period of the Roman Empire in the area of interest. The bigger the circle is, the more inscriptions share those coordinates. Sources: Natural Earth [31]; Ancient World Mapping Center (AWMC) [32]—Reprinted from AWMC database of shapefiles [32] under a CC BY license, with permission from AWMC, original copyright 2012; Epigraphic Database Heidelberg [29]; Dataset of the variables [49].

Inscriptions naming Apollo (N = 195), Minerva (N = 194), and Jupiter (in the form of *Iovi Optimo Maximo*, N = 2187) were selected based on the same rationale from the EDH as the inscriptions for Asclepius; i.e., to approximate spatially the worship and the habit of making inscriptions to them [49]. The spatial worship of these deities was included to avoid a potential caveat. Since Roman military structures and encampments are among the most frequently excavated objects from the era [60, 61], inscriptions dedicated to Roman deities are often found in these contexts. There is thus an inherent bias to the data, rendering many of the cults in the provinces military. However, if the spatial distribution of the worship of these deities differs despite this bias, we can assume that there were other factors at play (see Fig 3). The selection of these additional cults aims to represent varying aspects and dynamics potentially involved in their dissemination. The cult of Apollo was partially related to that of Asclepius, as Apollo is a mythical father of Asclepius, and although its aspects are universal, his cult is in some contexts related to healing as well [62]. A partial spatial overlap with the cult of Asclepius in the area of interest is thus to be expected. The cult of Minerva is representative of a cult with strong ties to the military. One of the attributes of Minerva is as the goddess of strategic warfare [62] and an entire legion, *Legio I Minervia*, founded by the emperor Domitian, was devoted to her, and the shield-covers of its soldiers bore depictions of the goddess [63, 64]. Inscriptions containing *Iovi Optimo Maximo* were selected as a proxy for the spatial dissemination of the worship of Jupiter. The cult of the chief deity in the Roman pantheon [62] is included as a type of “null-hypothesis” because it is expected that inscriptions dedicated to Jupiter were so widespread in the provinces that they spatially covered most of the Roman presence there.

As a final variable, a geocoded dataset from AWMC [32] representing Roman settlements in the provinces of interest (N = 621, places also searchable in the Pleiades database of ancient places [65]) was selected as an alternative proxy to the military presence for exploring spatial relationships in the dissemination of the selected cults and to test whether the cults were more spatially attached to military than settlement contexts. The settlements were filtered and incorporated according to the following criteria: 1) location is coded as certain, 2) type of the feature is settlement, and 3) settlement is attested for Roman period [49].

From the observation of the spatial dissemination of inscriptions in the provinces of interest, a few notable differences between the cults appear. First, the cult of Asclepius is the only cult from the selection that is not represented on the Antonine wall in Britannia. Second, the cult of Asclepius is also the only cult without inscriptions in Raetia. When compared to the other cults, the cult of Asclepius does not appear in bigger numbers in the central territory of Germania Superior. Minerva is represented consistently along the whole border in the area of interest, which could have been related to her popularity among soldiers. The cult of Apollo is spatially dominant in the southern parts of Germania Superior and the cult of Jupiter is, as expected, the most widespread.

This initial spatial comparison already proves relevant as it highlights differences in the spatial dissemination of these cults, and it will be possible to pursue these differences in future case studies. One future direction might involve the exploration of the case in Raetia where the cult of Asclepius is absent from the material evidence. The reason might be that a specific regional dynamic was involved in the form of a local healing deity; i.e.,—Apollo Grannus (with Grannus being of Celtic origins) [64, 66]. Cassius Dio writes in his *Historia Romana* (78.15.3–7) that Emperor Caracalla sought help from a trio of healing deities: Asclepius, Sarapis, and Apollo Grannus. The emperor even visited the temple of Apollo Grannus in Raetia in 213 CE [2, 67–69]. An inscription dated to the era of the Roman Empire dedicated to Apollo Grannus and Hygiea is also attested in Raetia. Hygiea is a mythical daughter of Asclepius [5]. It is thus plausible to hypothesize that the local Apollo Grannus assumed the role of the god of medicine elsewhere attributed to Asclepius.

Before proceeding to the spatial proximity analyses based on the distances measured on the transportation network, some remarks on the collection and the use of the data from EDH are necessary [29]. The EDH dataset is representative for the provinces of interest but certainly not for the whole area of the Roman Empire. Inscriptions from some of the regions are collected and stored in different databases such as Epigraphic Database Roma (EDR) [70], responsible for the area of Italy, or Hispania Epigraphica (HE) for Spain [71]. The visualizations presented in this article as well as the N of variables stated in the text are related to inscriptions in the provinces of interest. In the spatial proximity analyses, however, inscriptions from neighboring provinces were also included, in order to allow for the reality that the area of interest was not a series of islands separated from their surrounding contexts [49]: excluding potentially spatially related variables only because they were located just outside the borders of the provinces of interest would be methodologically flawed.

### Worship of Asclepius among roman soldiers–Spatial proximity analysis

**Fig 4:**
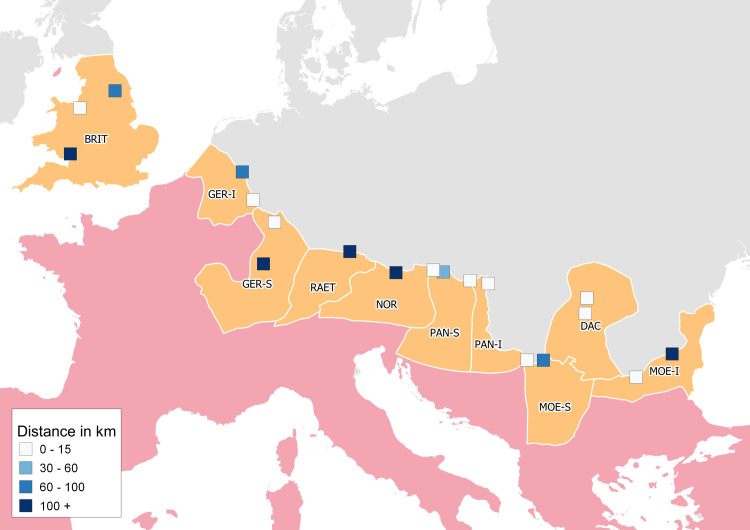
Long-term legion bases in the area of interest and their distance in the transportation network to their nearest proxy for the worship of Asclepius. Distance ranges are indicated in the legend. Sources: Natural Earth [31]; Ancient World Mapping Center (AWMC) [32]—Reprinted from AWMC database of shapefiles [32] under a CC BY license, with permission from AWMC, original copyright 2012; Dataset of the variables [49].

The first analytical task was to explore whether the worship of Asclepius, and the other deities, appeared in military contexts in the provinces of interest and whether the overlap was only occasional or if it appears across the provinces. To determine if the worship of Asclepius appeared in the proximity of long-term legion bases, a GIS analysis measuring the distances (in meters) on the transportation network from each legion base to its nearest proxy (i.e., inscription) for the worship of Asclepius was conducted. Only temporally possible connections, i.e., between proxies with overlapping dates, were allowed. The results are visualized in Fig 4. Because the distance of the inscriptions from the nearest Roman road differs, a simple rule determining whether a variable was still part of the transportation network or not was implemented. A threshold of 10 kilometers from the road was selected based on observations and testing in GIS software, as the variables within this threshold were still disseminated visibly along or at the ends of specific Roman roads, and variables beyond this threshold were either no longer attributable to a certain road or were completely outside the network. This rule is used in this and all subsequent spatial proximity analyses based on the distances on the transportation network presented here.

From the results visualized in Fig 4, it can be observed that the occurrence of the cult of Asclepius at or near the long-term legion bases is common in most of the provinces of interest except for Raetia and Noricum. Moreover, there are three sanctuaries of Asclepius directly in Roman settlements with long-term legion bases in Brigetio, Aquincum (Pannonia), and Apulum (Dacia) [5]. Additionally, in Aquincum, Carnuntum, Vindobona (Pannonia), and Novae (Moesia Inferior), the worship of Asclepius is attested in legionary hospitals (*valetudinaria*) [2, 5].

This measurement was repeated also for the cult of Apollo, Minerva, and Jupiter to compare and contextualize the spatial patterns. The medians of the distances from the long-term legion bases to the nearest individual cult proxies are as follows (in meters): Asclepius (11583), Apollo (4449), Minerva (835), Jupiter (111). Next step was to compare the patterns for specific cults further statistically. Since the distribution of the distances is non-normal (based on the Shapiro-Wilk test), a nonparametric rank based Kruskal-Wallis H test was used to test whether the measured distances for individual cults originate from the same distribution. The test revealed that the difference between the mean ranks of distances for individual cults is statistically significant: *H*(3) = 15.50608 *p* = 0.0014. The test does not identify between which cults the difference occurs. Therefore, a Mann-Whitney U test was used to compare all pairs of the four groups (i.e., distances from legion bases to proxies for four deities—Asclepius, Apollo, Minerva, and Jupiter). This test merges the data from two tested groups, sorts the data by value into ranks and compares the probability to get higher value from group A with the probability to get higher value from group B. The test revealed that the mutual differences between randomly selected values of all groups were not significant with one exception–Jupiter. The difference in the ranks of distances from legion bases to the nearest proxy for the cult of Jupiter when compared to all other cults was always significant.

These results allow for several observations. Based on the medians of distances, it can be inferred that in overall, the long-term legion bases had the cult of Jupiter and Minerva in proximity to a greater extent than they had the cults of Asclepius and Apollo. However, informed by the subsequent statistical tests, the proximity from legion bases to Asclepius, Apollo, and Minerva is still comparable while the proximity to Jupiter is significantly different. To put it in historical context, these observations show that the cult of Jupiter was widespread and established in many legion bases, the cult of Minerva is represented near legion bases to a large extent despite a significantly lower number of inscriptions than those attested for Jupiter in the area of interest. In this regard, the cult of Minerva probably benefitted from aspects related to warfare. Finally, the cult of Asclepius and Apollo are often attested near legion bases but were not spatially as widespread in this environment (Asclepius particularly) as the other cults.

**Fig 5:**
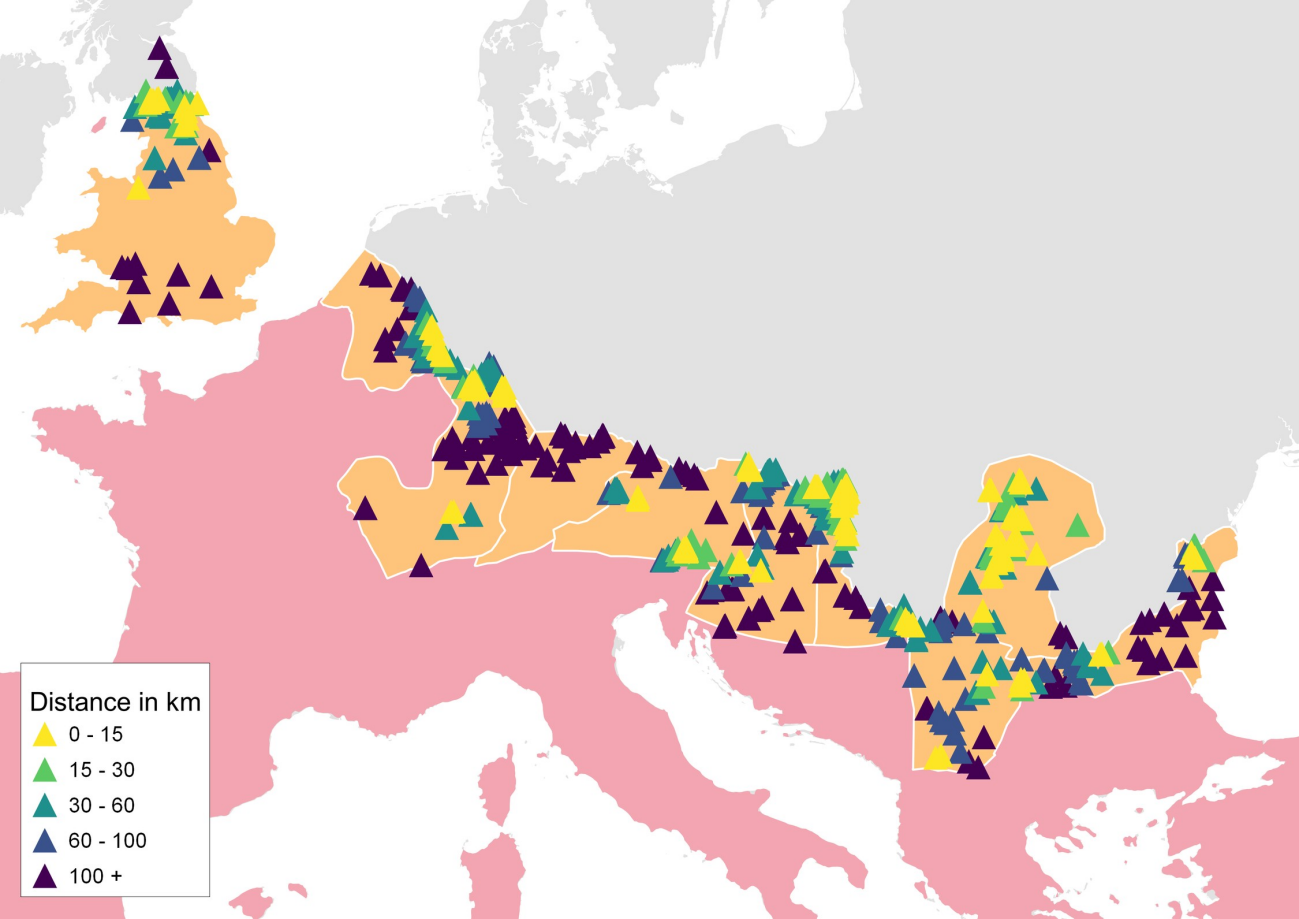
Locations of soldiers engaged in the practice of making votive inscriptions from the time of the Roman Empire and their distances in the transportation network to the nearest proxy for the worship of Asclepius in the area of interest. Distance ranges are indicated in the legend. Sources: Natural Earth [31]; Ancient World Mapping Center (AWMC) [32]—Reprinted from AWMC database of shapefiles [32] under a CC BY license, with permission from AWMC, original copyright 2012; Dataset of the variables [49].

Although this analysis of the distances, contents and contexts of inscriptions reveals a positive and, in some cases, even institutionally interconnected spatial relationship between the military environment and the worship of Asclepius as well as the other selected deities and does not contradict the argument that the Roman army had an impact on the spatial dissemination of the cult, the proxy for military presence is not sufficiently dense to reveal more complex spatial trends. As was already described above in the section on variables, the geocoded EDH votive inscriptions involving military personnel is a more granular and denser, and thus more appropriate proxy than legion bases for this task. As in the previous section, a spatial proximity analysis measuring the shortest distances on the transportation network between the location of soldiers and their nearest proxies for the worship of Asclepius was conducted in the first step. The results are visualized in Fig 5.

The positive spatial relationships between the locations of soldiers and the worship of Asclepius are visible in the majority of provinces. The pattern is visually particularly strong along the outer border of the Roman Empire. Again, to gain a deeper understanding in this regard, this spatial proximity analysis was conducted also for distances from soldiers to the other cults. Furthermore, to help determine whether the worship of these cults was indeed inherent to Roman soldiers, an alternative set of locations for the measurements of distances to the nearest cult proxies was introduced in the form of Roman settlements. A Mann-Whitney U test was applied to compare distances from soldiers to a cult with distances from settlements to that same cult. As was explained above, the Mann-Whitney U test sorts the distances based on ranks (keeping the information to which group a distance belongs, the shortest distance with rank 1, the longest distance ranked as the last) and tests the null hypothesis that the distributions of both groups are identical, i.e., that there is a 50% probability that an observation from a value randomly selected from one group exceeds an observation randomly selected from the other group. If the H_0_ is rejected, it means that the random observations are significantly different. The problem is that many of the votive inscriptions made by Roman soldiers have the same coordinates or appear in close spatial clusters within a Roman settlement, thus introducing a statistical blur to the analysis that would count each inscription from these clusters as a unique military position while in historical reality, these inscriptions represented identical spatial contexts. After test measurements in GIS software, a rule was introduced to eliminate this problem. Groups of inscriptions made by Roman soldiers (2 inscriptions or more) with the mutual distance between nearest neighbors equal to or under 3 kilometers were transformed into one central point (centroid). The introduction of this rule resulted in a new set of geocoded points representing unique spatial positions of Roman soldiers that could be implemented to the analysis without the risk of one military location being counted more than once, which would have produced statistical noise [49]. The results of the Mann-Whitney U test comparing the distances in the provinces of interest to the nearest cults from these military centroids (N of measured connections = 441) and from Roman settlements (N of measured connections = 593) are listed in Table 1. The N of measured connections is in some cases slightly lower than is the N of the variable in the dataset because not every variable is located in the proximity of the road network, or they are located on an isolated road that is for example connecting military structures but not settlements.

**Table 1:** Mann-Whitney U test results for distances from military positions and Roman settlements to their nearest place of worship of Asclepius, Apollo, Minerva, and Jupiter.

Variable	Rank Sum	Rank Sum	U	Z	*p*-value	Median	Median
						military	settlements
						(meters)	(meters)
	military	settlements					
To Asclepius	181527.5	353567.5	84066.50	-9.83089	<0.001	45505.25	99052.65
To Apollo	192083.0	343012.0	94622.00	-7.60834	<0.001	29391.24	57580.31
To Minerva	184746.5	350348.5	87285.50	-9.15310	<0.001	26043.90	51702.42
To Jupiter	148696.0	386399.0	51235.00	-16.7439	<0.001	155.72	14352.23

The results in Table 1 reveal that the null-hypothesis for ranked distances from military positions when compared to the ranked distances from settlements to each cult was rejected based on the *p*-value (well below 0.05). As a rule, the test ranks the short distances higher and the long distances lower. The distances from military positions scored higher than the distances from settlements to each cult and the test indicated that this difference was significant. Based on the scores and medians, it is possible to state that across the provinces of interest, the military positions had each of the cults significantly closer than was the case for settlements.

With respect to differences between the cults, the situation is similar to the distances for legion bases. Again, the cult of Asclepius was not as spatially widespread as the other cults and therefore, there were some military positions (for example in Raetia, where the cult was absent) that had the cult in greater distance than in the case of the other cults. This analytical step allowed for answering the question to which extent the soldiers had the cults in greater proximity than settlements had, and the statistically supported answer is that to a significant extent. This thus helps increase the validity of the argument that the worship of the selected cults was related to the social environment of the Roman army across the provinces.

**Fig 6:**
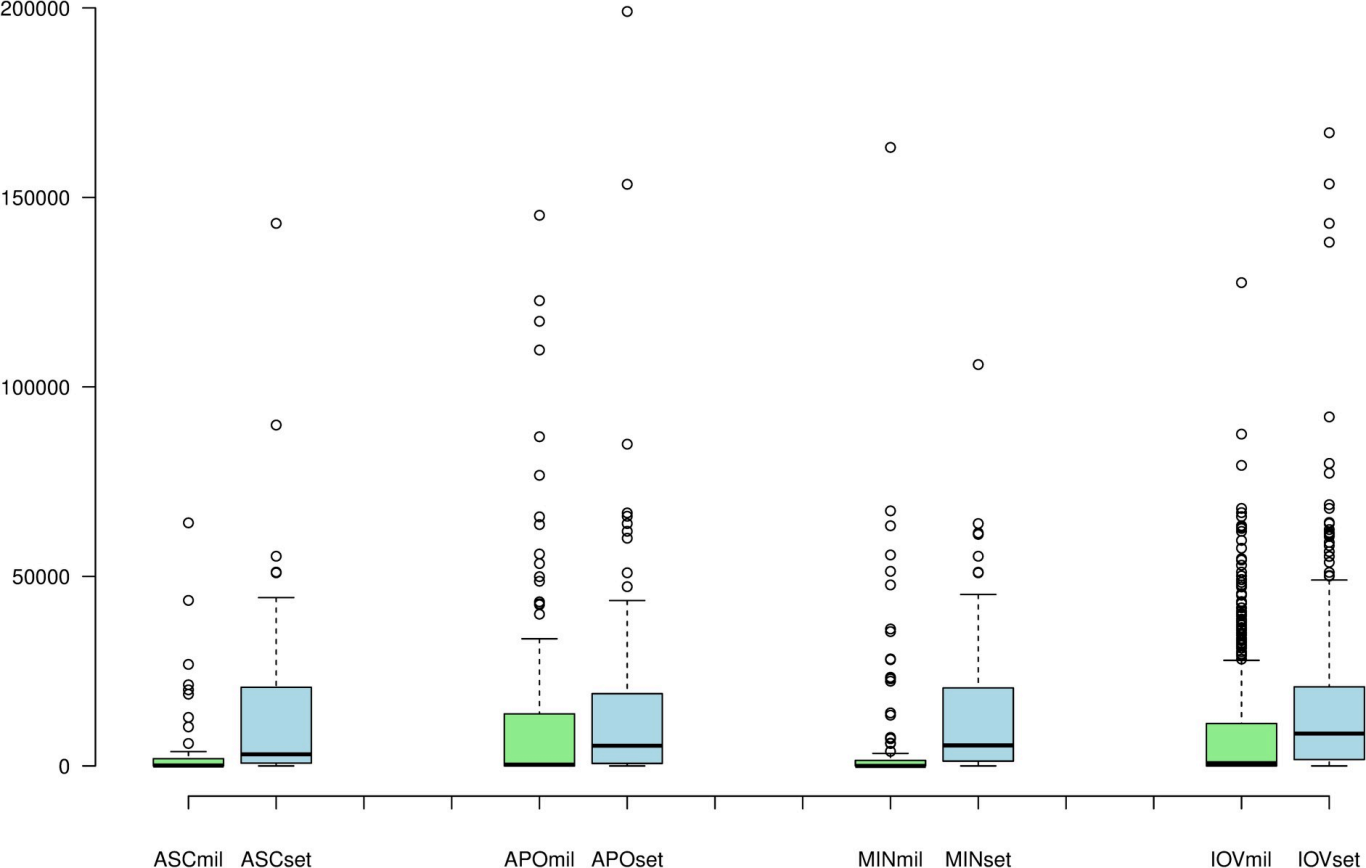
A box plot of distances measured on the transportation network (in meters) for all provinces of interest from unique cult locations to military proxies and Roman settlements. X axis shows labels for measurements with respect to individual cults. ASC (Asclepius), APO (Apollo), MIN (Minerva), IOV (Jupiter); mil (distances to the nearest military proxy), set (distances to the nearest settlement). Y axis shows the length of the distance reached in meters. Box plot: center lines show the medians; box limits indicate the 25th and 75th percentiles; whiskers extend 1.5 times the interquartile range from the 25th and 75th percentiles, outliers are represented by dots, their increased amount is caused by non-normal distribution of the data. Sources: Dataset of the variables [49].

The next step was to conduct a methodologically similar proximity analysis, but this time, in reverse order, i.e., evaluating distances measured **from** the cult places **to** their nearest military position defined by the soldiers dedicating votive inscriptions and to their nearest Roman settlement. This way, it was possible to discern in proximity to what these cult sites were actually located. In other words, this analysis helps explore whether the cult of Asclepius, despite its scarcer spatial spread, was more frequently located in military locations or not and how the pattern for Asclepius fits into the picture when compared with the other cults. As in the case of military positions, the analysis is focused on evaluating the dissemination of unique cult positions and therefore the geocoded inscriptions for individual deities that shared the same coordinates were transformed into centroids based on the 3-km to nearest neighbor rule to represent each location only once for each cult, thus avoiding duplicities and statistical blurs. Before evaluating the distances by groups using a Mann-Whitney U test, a box-plot graph was created to make initial observations of the patterns in the distances (Fig 6).

From the graph, it is clearly observable that the cults were often located directly at or near military positions and more so than in the case of settlements. Particularly the cult of Asclepius and Minerva, with the exception of some outliers, have the majority of distances to the nearest military proxy close to zero. The cult of Apollo and Jupiter have more variability in the distances, one explanation might be that these were universalistic deities with an appeal to a broader audience. The important indication from the graph for the research problem of this article is that it suggests that the cult of Asclepius was strongly attached to the military environment. The significance of the differences between the ranks of the distances to the nearest military positions and Roman settlements was confirmed for each cult by Mann-Whitney U test (Table 2). The scores and the medians again support the argument that all the cults were more spatially connected to military context than they were to settlements. Again, the slight difference between the N of measured distances is related to few cases of an isolated road connecting military locations without reaching a settlement.

**Table 2:** Mann-Whitney U test results for distances from cult locations to their nearest military position and Roman settlements.

**Variable**	**Rank Sum (military)**	**Rank Sum (settlements)**	**U**	**Z**	**p-value**
From Asclepius	1658.000	2713.000	530.0000	-4.23015	<0.001
From Apollo	10120.00	13533.00	4125.000	-3.80692	<0.001
From Minerva	7309.000	13397.00	2056.000	-7.39444	<0.001
From Jupiter	237360.5	343142.5	90749.50	-10.6268	<0.001
**Variable**	**Median**	**Median**	**Valid N (military)**	**Valid N (settlements)**	
	**military**	**settlements**			
	**(meters)**	**(meters)**			
From Asclepius	88.844	3059.116	47	46	
From Apollo	335.450	5323.494	109	108	
From Minerva	6.135	5402.641	102	101	
From Jupiter	721.304	8542.013	541	536	

The interpretation of the results yielded by these spatial proximity analyses together with the information from the epigraphic and archaeological evidence is that the practice of naming Asclepius was indeed tied to the military environment. The observation that military positions did not always have the cult of Asclepius within reach when compared to the other cults is explainable by the historical reality that the cult of Asclepius was not as widespread in some of the provinces and is attested by a lower number of inscriptions. A contributing factor to this could have been the specific orientation on health while the other cults were more universal. The question of how the healing aspect of the cult of Asclepius could have been related to a specific social context within the military is examined in the following section.

### Roman physicians—Spatial proximity analysis and correlation

**Fig 7:**
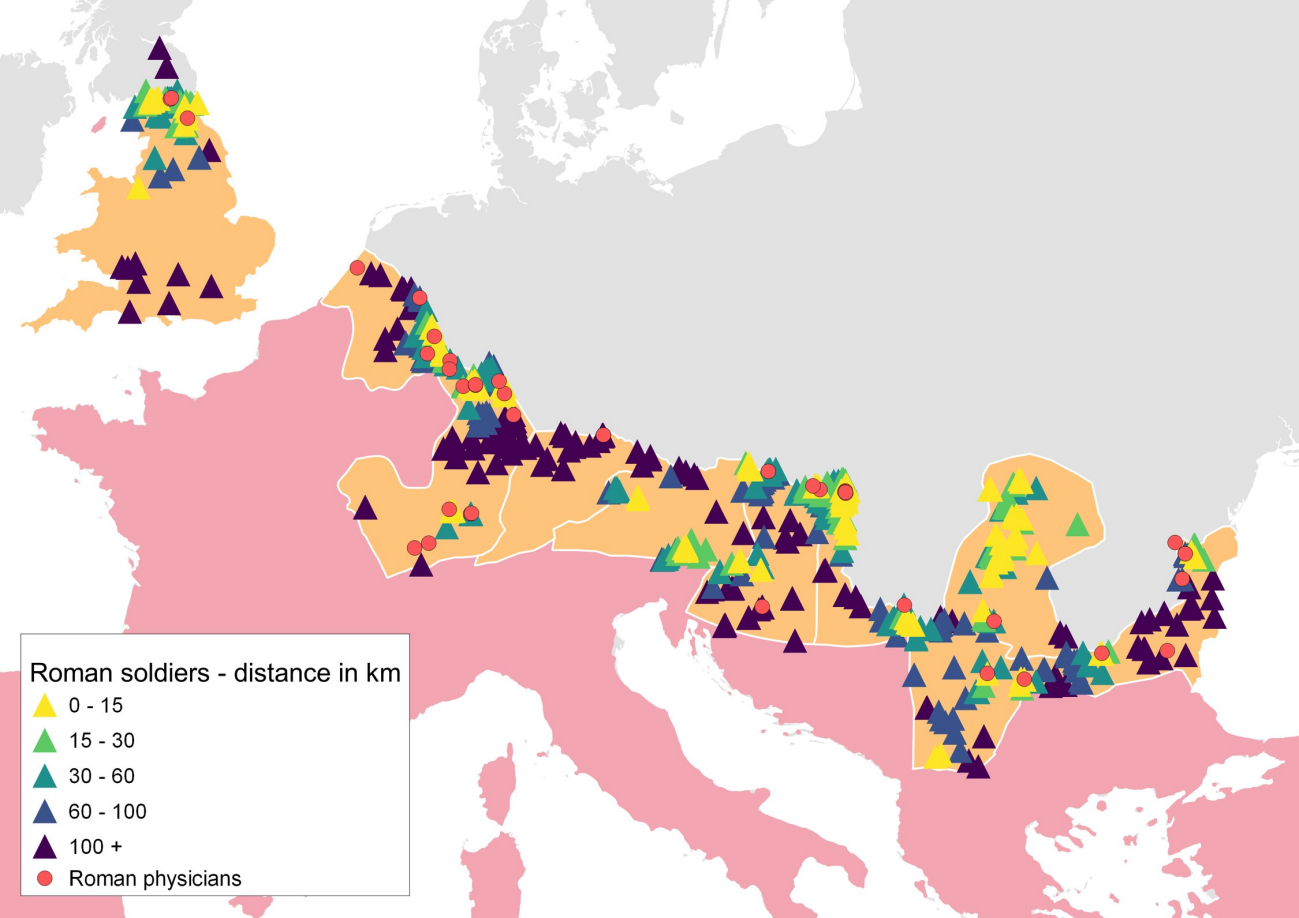
Attested locations of physicians in the context of the military environment and its proximity to the worship of Asclepius. Distance ranges between the Roman soldiers and the worship of Asclepius are indicated in the legend. Sources: Natural Earth [31]; Ancient World Mapping Center (AWMC) [32]—Reprinted from AWMC database of shapefiles [32] under a CC BY license, with permission from AWMC, original copyright 2012; Dataset of the variables [49].

The spatial analyses described in the previous section thus support the argument forwarded by van der Ploeg and others that soldiers participated in the cult of Asclepius, this time from the perspective of data analysis. This allows us to hypothesize that the social environment of the Roman Army represented a favorable factor for the spread of the cult of Asclepius. The working hypothesis for the following spatial proximity analysis is that the spatial traces of Roman physicians are a relevant predictor for the spatial occurrence of the worship of Asclepius since they are indicators of activities related to health risk and treatment; i.e., activities compatible with the healing aspects attributed to Asclepius. The first step in testing this hypothesis was to visualize the relevant variables and inspect visible patterns (Fig 7).

Fig 7 demonstrates that the location of physicians in the military environment in the provinces of interest frequently appears in similar spatial contexts as the worship of Asclepius. However, this hypothesis involves three variables (soldiers, worship of Asclepius, physicians) which brings more complexity to the relationship analysis of the transportation network, and with each added variable it is increasingly difficult to evaluate these relationships by visualizing their spatial distribution. Moreover, to further explore the question of whether there was a more intensive inclination to worship Asclepius in places where physicians were active and mentioned more frequently in inscriptions than elsewhere, the other selected cults needed to be added into the analysis as well. A statistical analysis using the Spearman correlation of the shortest distances (in meters) on the Roman roads from the positions of votive inscriptions made by Roman soldiers to their nearest inscription with Asclepius and to their nearest inscription mentioning the occupation of a physician was conducted to explore whether these relationships in the environment of the Roman army are also significant from a statistical perspective. The shortest distances from soldiers’ locations to spatial proxies for the worship of Apollo, Minerva, and Jupiter on the transportation network were subsequently included in the analysis in the same manner as the distances to the proxies for the worship of Asclepius. The Spearman rank correlation coefficient (*R*_*s*_; always between -1.0, i.e., a perfect negative correlation, and 1.0, i.e., a perfect positive correlation) measures the strength, or “tightness”, and direction of the association between two ranked variables and is suitable for evaluating correlations where the variables do not possess a normal (Gaussian) distribution, which is the case for the distribution of the measured distances in this research. In other words, the Spearman correlation allows us to check if there is a significant positive correlation between the ranks of distances in the road network between Roman soldiers and physicians on the one hand, and between Roman soldiers and the worship of Asclepius on the other. This spatial analysis asks the same question for each location of a Roman soldier; i.e., how far (in meters) from that location (using a Roman road) is the nearest proxy for a physician and how far is the nearest proxy for the worship of Asclepius. If these distances rank similarly on the scale of a province, then the correlation is positive.

The statistical analysis was conducted in two formats. First, the distances from the positions of Roman soldiers to their nearest proxies of cults and physicians were measured all together without incorporating the dates of origins for these variables, the only temporal attribute being that they originated in the time of the Roman Empire [49]. This preliminary analysis was conducted to observe initial spatial trends in the data. In the second step, more specific temporal attributes were introduced (for a similar two-step approach to spatial data with respect to temporal aspects see e.g., [8]). However, it was not possible to simply use all the votive inscriptions made by Roman soldiers in both steps of this analysis as the soldiers’ geographical positions for the transportation network measurements. The question of interest is how unique locations with the presence of Roman soldiers and their relationship to spatial occurrences of Roman physicians relate to the spatial occurrence of the worship of Asclepius. For this reason, the military inscriptions sharing coordinates were again transformed into centroids representing each military position only once as a unique location identically as was the case in the methodological section focusing on the role of soldiers [49]. To have some idea about the spatial proportionality of Roman physicians based on the inscriptions, their numbers were compared to the unique positions of Roman soldiers. For 7 out of 10 provinces of interest (the exceptions being Noricum, Dacia, and Moesia Superior), the number of inscriptions attesting the presence of Roman physicians ranges between 5–16% of the amount of unique positions of Roman soldiers. Moesia Superior fits into this pattern as well with physicians attested just at the borders. This means that the spatial proportionality of the proxy for Roman physicians is consistent across provinces without dramatic spikes, which is a positive attribute for statistical evaluation. In Dacia and Noricum, there are almost no physicians attested in the inscriptions and it is therefore expected that the statistical results from these two provinces show no or only very limited correlation. The results of the first step of analysis (outside of date ranges) are summarized in Table 3 and discussed in the Results section.

**Table 3:** Spearman correlation (*R*_*s*_) between distances from positions of Roman soldiers to their nearest proxy for the presence of a Roman physician and their nearest cult inscription in the era of the Roman Empire.

Provinces	*R*_*s*_ for individual cults			
	Asclepius	Apollo	Minerva	Jupiter
Britannia	0.665	0.577	0.582	0.244
Germania Inferior	0.483	0.288	0.564	0.316
Germania Superior	0.827	0.298	0.370	-0.017
Raetia	-0.097	-0.582	-0.124	0.412
Noricum	-0.282	-0.059	-0.063	-0.033
Pannonia Superior	0.362	0.524	0.649	-0.156
Pannonia Inferior	0.687	0.734	0.377	0.063
Moesia Superior	0.565	0.054	-0.464	-0.239
Moesia Inferior	0.539	0.088	-0.489	-0.024
Dacia	-0.224	-0.034	0.331	-0.130
*p*-value for positive correlations		*p*<0.05	*p*<0.01	*p*<0.001
*p*-value for negative correlations		*p*<0.05	*p*<0.01	*p*<0.001

*R*_*s*_ is always between -1.0, i.e., a perfect negative correlation, and 1.0, i.e., a perfect positive correlation. With respect to *p*-values, *p*<0.05 is significant, *p*<0.01 and *p*<0.001 are highly significant.

The second step of the statistical analysis, which evaluated the correlation between the spatial occurrence of Roman physicians in inscriptions and the worship of Asclepius from the perspective of soldiers’ positions within the transportation network in the provinces of interest, involved a temporal aspect by allowing connections only between temporally related variables. However, a large portion of the EDH [29] inscriptions has a gap of 100 years between the *terminus post* and *ante quem*, and the majority of the inscriptions used in this research have ranges of at least 50 years for their possible dates of origin. This introduces significant temporal uncertainty to the analysis. Because the inscriptions are often dated only on the level of whole centuries, the data were given attributes to reflect this quality; i.e., each inscription carries information about which century or centuries it can be dated to [49]. The statistical analysis then evaluated the correlations between the variables for individual centuries separately to observe the potential trends in possible temporal contexts. Because the vast majority of the data is from the 2nd and 3rd century CE, and the amount of data from the 1st centuries BCE and CE, as well as the 4th and 5th centuries CE, is marginal and thus unsuitable for statistical evaluation [29]: only the data that were possible to date to the 2nd and 3rd century CE were included in the temporal analysis [49]. With respect to the dating of inscriptions attesting Roman physicians in the provinces of interest, approximately 77% (N = 33) can be dated to the 2nd century CE and 26% (N = 11) are dated exclusively to the 2nd century; 63% (N = 27) can be dated to the 3rd century CE and 12% (N = 5) belong exclusively to the 3rd century CE. Also, 88% (N = 38) of these inscriptions have their *terminus post quem* with respect to dating earlier than 200 CE. Therefore, the data suggest that the habit of mentioning Roman physicians in inscriptions was possibly stronger in the 2nd century CE than in the 3rd. As in the first (cross-period) step of the analysis, the positions of Roman soldiers were transformed into centroids carrying the temporal information of the clustered inscriptions based on the 3-kilometers-between-nearest-neighbors rule to represent each unique spatial position [49]. The results from this second step of the analysis are summarized in Table 4 and elaborated in the Results section.

**Table 4:** Spearman correlation (*R*_*s*_) between distances from positions of Roman soldiers to their nearest proxy for the presence of a Roman physician and their nearest cult inscription, based on the data that can be dated to the 2nd and 3rd century CE.

**2nd century CE time frame**				
**Provinces**	***R***_***s***_ **for individual cults**			
	**Asclepius**	**Apollo**	**Minerva**	**Jupiter**
Britannia	0.805	0.650	0.625	0.165
Germania Inferior	0.275	-0.072	-0.180	0.177
Germania Superior	0.760	0.284	0.286	-0.027
Raetia	0.029	-0.704	-0.821	0.323
Noricum	-0.101	0.135	0.474	0.209
Pannonia Superior	0.504	0.677	0.702	0.044
Pannonia Inferior	0.835	0.702	0.398	-0.190
Moesia Superior	0.549	-0.131	-0.581	-0.384
Moesia Inferior	0.632	0.271	-0.593	0.047
Dacia	-0.295	-0.041	0.088	-0.142
**3rd century CE time frame**				
**Provinces**	***R***_***s***_ **for individual cults**			
	**Asclepius**	**Apollo**	**Minerva**	**Jupiter**
Britannia	0.659	0.626	0.593	0.316
Germania Inferior	0.248	0.353	0.444	0.348
Germania Superior	0.818	0.316	0.053	-0.123
Raetia	0.344	-0.612	-0.018	0.274
Noricum	-0.381	0.109	0.006	0.227
Pannonia Superior	0.521	0.714	0.807	-0.056
Pannonia Inferior	0.686	0.743	0.282	-0.127
Moesia Superior	0.739	0.103	-0.486	-0.259
Moesia Inferior	0.096	-0.050	-0.130	0.018
Dacia	-0.310	-0.138	0.073	-0.195
*p*-value for positive correlations		*p*<0.05	*p*<0.01	*p*<0.001
*p*-value for negative correlations		*p*<0.05	*p*<0.01	*p*<0.001

*R*_*s*_ is always between -1.0, i.e., a perfect negative correlation, and 1.0, i.e. a perfect positive correlation. With respect to *p*-values, *p*<0.05 is significant, *p*<0.01 and *p*<0.001 are highly significant.

## Results

The results of the spatial proximity analysis based on the distances measured on the transportation network support the hypothesis proposed in the academic discussion that soldiers in antiquity were significant facilitators of the spread of ancient cults to new socio-spatial environments. The results also support this hypothesis in its more specific form, claiming that Roman soldiers had a positive impact on the spread of the cult of Asclepius. The spatial proximity analysis exploring the relationships between the long-term bases of Roman legions and proxies for the worship of Asclepius in the era of the Roman Empire revealed that the worship of Asclepius commonly appeared in the proximity of such bases in the majority of the provinces of interest comparably as the cults of Apollo and Minerva. The spatial distribution of the cult of Jupiter stands out as the most widespread. In some cases, this relationship was directly tied to a legion base or a military hospital within a base, which is considered in the academic discussion as an indicator that healing in the environment of Roman legions was considered a dual responsibility of physicians and deities with attributes related to medicine and that there was no conceptual division forcing the wounded soldiers to choose between divine and “human” medicine [2, 57].

The spatial proximity analysis focusing on the proximity of the worship of Asclepius to the positions of Roman soldiers based on the votive inscriptions also confirms these spatial trends. When compared to the spatial distribution of the cults of Apollo, Minerva, and Jupiter, the cult of Asclepius was not as widespread in the provinces of interest, i.e., although the attested cult was in majority located in military environments, not every military position had the cult of Asclepius in their proximity. Visual inspection of the data on the map suggests that the relationship between these two variables is particularly strong at the outer borders of the Roman Empire in the majority of the provinces of interest, the only exception being Raetia where there is no evidence for the explicit worship of Asclepius.

The statistically evaluated spatial proximity analysis of the relationships between the proxies for the worship of Asclepius and Roman physicians in the context of Roman military environments yielded significant results (Tables 3 and 4). The first step of the statistical analysis, i.e., geographical with no detailed dating involved (using only proxies datable to the era of the Roman Empire; see Table 3), was conducted for initial observations of potential trends that could be expected in the second step. In the first step, the analysis revealed a significant positive correlation between the proxy for the worship of Asclepius and Roman physicians with respect to the distances from the positions of Roman soldiers on the transportation network in 7 out of 10 provinces (i.e., Britannia, Germania Superior and Inferior, Pannonia Superior and Inferior, and Moesia Superior and Inferior). This result suggests that Roman physicians and the worship of Asclepius appeared in a similar Roman military environment across the provinces of interest. The lack of highly significant correlations in Raetia, Dacia, and Noricum was expected as there is no proxy for Asclepius in Raetia, and Dacia and Noricum have almost no documented physicians. Moreover, when compared to other cults, the correlation between Roman physicians and the spatial worship of Asclepius is stronger, more significant, and appears in more provinces, with the exception of Germania Inferior, which has a stronger correlation for Minerva, and Pannonias, where Apollo and Minerva have comparable or stronger correlations. The partial overlap of Apollo and Minerva is understandable, as Apollo shares some aspects with Asclepius and Minerva frequently appears on the outer border of the Roman Empire in the provinces of interest, as well as in legionary bases where physicians are attested. No positive correlation for the worship of Jupiter was expected, as the cult was so widespread that it cannot be explained by the predictor of Roman physicians. Finally, Asclepius is the only correlated cult in the provinces Moesia Inferior and Superior. In these two provinces, the cult of Minerva is actually negatively correlated with Roman physicians. This means that whenever a proxy for the cult of Minerva was more distant from the position of a Roman soldier, the nearest proxy for Roman physicians is likely to be very close to that soldier, and vice versa. This means that in these two provinces, it is not possible to explain the spatial dissemination of the cult of Minerva by the presence of Roman physicians at all.

The second step of the analysis, i.e., connecting and then comparing the distances between variables based on the centuries they can be dated to, revealed similar trends in the data as the first step. For the 2nd century CE (Table 4), positive correlations for the cult of Asclepius can be attested for the same provinces as in the first (geographical) step of the analysis; here the sole exception is Germania Inferior, where there is no correlation this time. The cult of Apollo is also positively correlated in the same provinces as in the previous step, with only minor shifts in the tightness of the correlations. Similarly, the cult of Minerva follows the previously documented trend with the difference that it is no longer correlated with the Roman physicians in Germania Inferior and Dacia and is newly positively correlated in Noricum. Again, the cult of Asclepius is positively correlated in more provinces than the other cults. The correlations for this cult are highly significant, and the Spearman rank correlation coefficient (*R*_*s*_) is higher than 0.5 for each correlated province (N = 6). The cults of Apollo and Minerva are higher in the strength of the correlation in Pannonia Superior. The cult of Jupiter, as in the previous step, is without correlation with respect to the spatial occurrence of Roman physicians. The trends for the 3rd century CE are also similar as in the steps above, but with some important differences. The cult of Asclepius is still positively correlated across the provinces, but, when compared to the 2nd century CE, it is no longer correlated in Moesia Inferior. Additionally, the correlation for Germania Superior for the 3rd century is actually a false positive. There are no inscriptions with Asclepius in Germania Superior dated to the 3rd century, and the data there are correlated by the logic that if there is an inscription with a Roman physician approximately 100 kilometers from the position of a Roman soldier, then the nearest proxy for the cult of Asclepius is twice as far away (and in a different province). These instances are a good reminder that it is not possible to study such research problems in methodological isolation from established historiographical methodology, as both are needed in the input as well as the output phases of the analysis. The cult of Apollo is again correlated in much the same manner as in the previous steps, and the cult of Minerva too, with the exception that it is no longer correlated in Germania Superior and Noricum, but this time is correlated in Germania Inferior as in the geographical stage of this analysis. The cult of Apollo and Minerva are more strongly correlated than the cult of Asclepius in Pannonia Superior (in both 2nd and 3rd century CE), and in the 3rd century, the cult of Apollo is more correlated than the cult of Asclepius in Pannonia Inferior. The cult of Asclepius is negatively correlated in the 3rd century in Noricum and Dacia, which is of only very limited relevance as there are almost no inscriptions mentioning Roman physicians there. It seems that the correlation for the cult of Asclepius is stronger for the 2nd century CE compared to the 3rd; however, we must bear in mind that much of the data can be assigned to both centuries, and although the temporal aspect adds some clarity to the data compared to the purely geographical distribution, the temporal trends remain in the realm of “potential” rather than “exact”. On the other hand, some factors could explain this trend. As was already mentioned, the habit of making inscriptions involving Roman physicians was possibly more intensive in the 2nd century CE, and in some cases Roman soldiers active in the 3rd century could have utilized already existing material culture bearing inscriptions with Asclepius for their worship, such as altars from the previous centuries. Finally, the inscriptions mentioning Roman physicians also mention Asclepius in the same inscription in 6 cases, Apollo in 3, Jupiter in 2 and Minerva in 0, which supports the working hypothesis of this article that the spatial occurrence of Roman physicians in inscriptions is tied with the cult of Asclepius more intensively than with other cults.

The statistical analyses provided consistent results in both the geographical and temporal steps, offering relevant insights into the potential factors impacting the spread of the cult of Asclepius in the Roman Empire. Despite the fact that the Roman soldiers and their proximate social environment worshipped many deities [2], it seems that the worship of Asclepius benefited from the presence of Roman physicians in these environments more than other cults. The consistency and the statistical significance of the results from the provinces also suggest that the presence of physicians was one of the key favorable conditions for the success of the cult of Asclepius among soldiers stationed in the provinces, despite the fact that the physicians demographically and spatially constituted a minority. The argument based on the quantitative analysis presented here that Roman physicians are a relevant predictor for the spatial occurrence of the worship of Asclepius in the military environment brings a more detailed view to the social level of the spread of this cult and has the potential to enrich the academic debate and stimulate further research in this direction.

## Discussion

By using formal GIS approaches, this article sheds new light on the topic of the spatial dissemination of the cult of Asclepius in the era of the Roman Empire. After establishing by means of spatial proximity analysis that the cult of Asclepius frequently appeared in the social environment of the Roman army, the study statistically evaluated the relationships between positions of Roman soldiers and their nearest proxies for the worship of Asclepius, as well as the presence of Roman physicians, to explore the working hypothesis of this article; i.e., that the spatial traces of Roman physicians are a relevant predictor for the spatial occurrence of the worship of Asclepius in the environment of the Roman army because of the common trait of both physicians and the cult of Asclepius–a focus on health. In all steps the statistical analysis revealed highly significant correlations in the spatial relationships between the traces of Roman physicians and the worship of Asclepius. Overall, the cult of Asclepius is more correlated with the inscriptions mentioning Roman physicians than other cults, and Asclepius is explicitly mentioned next to Roman physicians in the same inscriptions more often than the other deities. The working hypothesis thus found support in the data analysis and we now have a more nuanced understanding of the dynamics involved in the spread of the cult of Asclepius. The testable argument that Roman physicians played an important role in this process of cultural diffusion adds a new element to the debate, which can be now subjected to further academic scrutiny.

In terms of historical recontextualization, the results expand important topics from historical debates. There is a long-standing consensus based on the rich epigraphic documentation that the Roman army was a mobile part of the Roman Empire deeply engaged in the worship of Roman deities. The research presented here details this understanding by revealing quantitatively that even though the military worship of specific deities is attested, there are differences in the extent and spatial distribution of this worship. Furthermore, the results suggest that the diversity in the worship of deities in the military context could have resulted from activities of specific social groups within the army defined, for example, by profession. In this case, the cult of Asclepius was not as spatially widespread in the tested provinces as the other, more universalistic, cults. However, the analyses pointed out that the cult was to a significant degree attached to the military environment and benefited from the presence of Roman physicians. With respect to the debate on this topic, the results suggest that the evidence of individual inscriptions concerned with health where Asclepius is mentioned next to a physician are representative of a spatial pattern of worship reaching across provinces.

The research presented in this article also brings into focus problems for future inquiries, such as what the specific regional dynamics involved in the provinces with a very low count of either inscriptions naming Asclepius or Roman physicians are, or why the cult of Apollo was able to compete with the cult Asclepius with respect to the relationship with physicians in some provinces but not in Moesia Superior and Inferior, where the cult of Asclepius is the only correlated one, or why the cult of Asclepius is not represented on the Antonine wall.

This article also demonstrates that the combination of the established methodology of historiography with formal approaches is fruitful and has significant potential to bring further detail to our understanding of the process of cultural transmission in antiquity. At the same time, it is important to be aware of the limitations involved in this type of analysis, such as incomplete historical data where a portion of the material evidence is either not yet excavated and cataloged or has been completely destroyed by the tides of time, and for the data that are cataloged, it is often difficult to establish their exact dates of origins. For these reasons, the statistical results in this research cannot be taken as an unbreakable scientific statistical proof; rather, it should be perceived as a statistically supported argument pointing to a highly probable trend in the spread of the cult of Asclepius.
